# Fecal carriage of extended-spectrum β-lactamase-producing *Enterobacteriaceae* in hospital and community settings in Chad

**DOI:** 10.1186/s13756-019-0626-z

**Published:** 2019-10-31

**Authors:** Oumar Ouchar Mahamat, Abdelsalam Tidjani, Manon Lounnas, Mallorie Hide, Julio Benavides, Calèbe Somasse, Abdoul-Salam Ouedraogo, Soufiane Sanou, Christian Carrière, Anne-Laure Bañuls, Hélène Jean-Pierre, Yann Dumont, Sylvain Godreuil

**Affiliations:** 1 Laboratoire de Bactériologie, Centre Hospitalier Universitaire de Montpellier, Montpellier, France; 2 MIVEGEC, IRD, CNRS, Université de Montpellier, Montpellier, France; 3 Service de laboratoire Hôpital de la Mère et de l’Enfant, N’Djaména, Chad; 4 Faculté de Médecine, Université de N’Djaména, N’Djaména, Chad; 5 7 Departamento de Ecología y Biodiversidad, Facultad de Ciencias de la Vida, Universidad Andrés Bello, Santiago, Chile; 6 Service de bactériologie-virologie, Département des laboratoires, Centre Hospitalier Universitaire Souro Sanou, Bobo Dioulasso, Burkina Faso; 7 Laboraoire Mixte International, DRISA, IRD, Montpellier, France

**Keywords:** ESBL, *Enterobacteriaceae*, Fecal carriage, Chad

## Abstract

**Background:**

Fecal carriage of extended-spectrum β-lactamase-producing *Enterobacteriaceae* (ESBL-PE) remains poorly documented in Africa. The objective of this study was to determine the prevalence of ESBL-PE fecal carriage in Chad.

**Methods:**

In total, 200 fresh stool samples were collected from 100 healthy community volunteers and 100 hospitalized patients from January to March 2017. After screening using ESBL-selective agar plates and species identification by MALDI-TOF mass spectrometry, antibiotic susceptibility was tested using the disk diffusion method, and ESBL production confirmed with the double-disc synergy test. The different ESBL genes in potential ESBL-producing isolates were detected by PCR and double stranded DNA sequencing. *Escherichia coli* phylogenetic groups were determined using a PCR-based method.

**Results:**

ESBL-PE fecal carriage prevalence was 44.5% (51% among hospitalized patients vs 38% among healthy volunteers; *p* < 0.05). ESBL-producing isolates were mostly *Escherichia coli* (64/89) and *Klebsiella pneumoniae* (16/89). PCR and sequencing showed that 98.8% (87/89) of ESBL-PE harbored *bla*_CTX-M_ genes: *bla*_CTX-M-15_ in 94.25% (82/87) and *bla*_CTX-M_-_14_ in 5.75% (5/87). Phylogroup determination by quadruplex PCR indicated that ESBL-producing *E. coli* isolates belonged to group A (*n* = 17; 27%), C (*n* = 17; 27%), B2 (*n* = 9; 14%), B1 (*n* = 8; 13%), D (*n* = 8; 13%), E (*n* = 1; 1.6%), and F (*n* = 1; 1.6%). The ST131 clone was identified in 100% (9/9) of *E. coli* B2 strains.

**Conclusions:**

The high fecal carriage rate of ESBL-PE associated with CTX-M-15 in hospital and community settings of Chad highlights the risk for resistance transmission between non-pathogenic and pathogenic bacteria.

## Introduction

Extended-Spectrum β-Lactamase-Producing *Enterobacterial* (ESBL-PE) have spread worldwide, and have become endemic in several countries since their first description in 1983 [1, 2]. Their diffusion is mainly attributed to ESBL-encoding genes that are often carried by mobile genetic elements, such as plasmids, that facilitate their dissemination [3]. *Enterobacterial* are commensal bacteria present in the intestinal tract of humans and various animals, [4] are an important reservoir of resistance genes, leading to ESBL-PE dissemination in communities. This might result in hospital and community infections if ESBL-encoding genes are acquired by pathogenic bacteria, or if commensal ESBL-PE become pathogenic [5, 6]. Indeed, colonization by ESBL-PE is one of the main risk factors for infection with antibiotic-resistant bacteria (ARB) [7]. These infections pose a great challenge due to the limited therapeutic options (including prolonged hospitalization) and the increased morbidity and mortality rates [8]. Therefore, they are particularly problematic in low-income countries [9].

Fecal carriage of ESBL-PE has been increasingly reported worldwide over the last decade. The highest ESBL-PE carriage prevalence has been described in Asia [10], whereas prevalence rates are lower in Europe and North America [11, 12]. Conversely, data on ESBL-PE fecal carriage in Sub-Saharan Africa are limited, and a few available studies reported a prevalence ranging from 6 (in Mauritania) to 66% (in Cameroon) in healthy volunteers and hospitalized patients [13, 14]. This small number of studies does not facilitate the identification of factors associated with this carriage in order to prevent and reduce the spread of ARB.

We recently reported a high prevalence of ESBL-PE containing the CTX-M-15 enzyme in clinical isolates from three major Chadian hospital [15] (48%). However, the rare studies on fecal carriage of ESBL-PE in Chad [16–19] and the absence of molecular data on ESBL-encoding genes limit our understanding of the genes and clones circulating among the Chadian population.

The aim of this study was to investigate the prevalence of ESBL-PE fecal carriage among hospitalized patients and healthy volunteers in Chad, and to identify the associated ESBL-encoding genes.

## Materials and methods

### Settings and bacterial isolates

Fresh stool samples were collected from 100 patients hospitalized for more than 48 h at the Mother and Child hospital, and from 100 healthy volunteers in the community (healthy volunteers were recruited from health staff and university students), from January to March 2017. This university teaching hospital is in N’Djamena, the capital city of Chad (1.5 million inhabitants), and is the reference mother-child hospital in Chad. It has a capacity of 261 beds (including an intensive care unit), with about 5000 admissions and 45,000 outpatients in 2016. People with diarrheal diseases were excluded from the study. This study was approved by the hospital ethics board and the Chadian Ministry of Public Health (No 676/PR/PM/MSP/SE/SG/DRGP/DRH/SGF/16). Informed written consent was obtained from all subjects and from at least one parent for each child before enrolment in the study. Only information about age, and sex was available.

### ESBL-PE detection, bacterial identification, and antimicrobial susceptibility testing

Briefly, 0.5 g of each fresh stool sample was suspended in 5 ml of sterile saline solution (0.9%) and 100 ml aliquots were plated on ESBL agar plates (bioMérieux, Marcy-l’Etoile, France). Plates were examined after 24 and 48 h of incubation at 37 °C. Bacterial isolates were identified using biochemical tests and then confirmed by matrix-assisted laser desorption ionization-time of flight (MALDI-TOF) mass spectrometry (Bruker Daltonics, Bremen, Germany). Antimicrobial susceptibility testing was performed using the disk diffusion method on Müller-Hinton agar and the clinical breakpoints recommended by the European Committee on Antimicrobial Susceptibility Testing (EUCAST) guidelines (Version 7.1) (http://www.eucast.org/clinical_breakpoints/). The following antibiotics were tested: amoxicillin, amoxicillin-clavulanic acid, ticarcillin, ticarcillin-clavulanic acid, piperacillin, piperacillin-tazobactam, temocilin, cephalexin, aztreonam, cefotaxim, ceftazidim, cefepime, cefoxitin, ertapenem, imipenem, gentamicin, tobramycin, netilmicin, amikacin, trimethoprim + sulfamethoxazole, nalidixic acid, ofloxacin, ciprofloxacin, levofloxacin, chloramphenicol, and fosfomycin. ESBL production was detected phenotypically with the double-disk synergy method [20]. In the case of high-level cephalosporinase production, the double-disk synergy test was performed using cloxacillin-supplemented medium (250 mg/L).

### Molecular characterization of β-lactamase-encoding genes

DNA was extracted from each isolate using the boiling lysis method. Briefly, one single colony was transferred in 100 μL of distilled water, heated at 100 °C for 10 min, and then centrifuged. The presence of the most common ESBL-encoding genes, including *bla*CTX-M (CTX-M group 1, 2, 8, 9 and 25), *bla*TEM, *bla*SHV and *bla*OXA-like, was tested by multiplex PCR using the previously described primers and conditions [21]. DNA samples from reference *bla*CTX-M, *bla*TEM, *bla*SHV and *bla*OXA-like-positive strains were used as positive controls. PCR products were visualized by electrophoresis (100 V for 90 min) on 2% agarose gels containing ethidium bromide. A 100 bp DNA ladder (Promega, USA) was used as marker size. PCR products were sequenced bidirectionally on a 3100 ABI Prism Genetic Analyzer (Applied Biosystems). Sequencing data were analyzed online using the BLAST tool available at the National Center for Biotechnology Information web page (https://blast.ncbi.nlm.nih.gov/Blast.cgi).

### Genotyping of ESBL-producing *E. coli* isolates

The phylogroup (A, B1, B2, C, D, E, and F) of each ESBL-producing *E. coli* isolate was determined using the quadruplex PCR-based method developed by Clermont et al. [22] For strains assigned to the B2 phylogenetic group, the presence of the *E. coli* sequence type (ST) 131 also was analyzed using a O25b-specific PCR method with pabB and trpA (control) allele-specific primers, as previously described [23].

### Statistics

Statistical analyses were performed using the Epi Info software, version 3.5.3 (Centers for Disease Control and Prevention, Atlanta, GA, USA). Differences in the proportion of ESBL-producers between patient groups were assessed using the Chi-square test. A *p*-value < 0.05 was considered as statistically significant.

## Results

### Characteristics of participants and ESBL-PE prevalence

During the study period, 100 healthy volunteers and 100 hospitalized patients (*n* = 115 women and *n* = 85 men; age: 1 to 54 years) were screened. The overall frequency of ESBL-PE fecal carriage was 44.5%. ESBL-PE rate was significantly higher among hospitalized patients (51%) than healthy volunteers (51% vs 38%; *p* < 0.05, Chi-square test). Carriage rate was not different between sexes and in the three age groups (Table 1).

**Table 1:** Demographic characteristics and prevalence of ESBL-PE carriage in the study population

Variable	Hospitalized patients (*n* = 51)	Healthy volunteers (*n* = 38)	Total (*n* = 89)
Sex			
Men, *n* (%)	15 (29%)	23 (60%)	38 (43%)
Women *n* (%)	36 (71%)	15 (40%)	51 (57%)
Age groups (years)			
1–11 *n* (%)	18 (35%)	3 (8%)	21 (24%)
12–24 *n* (%)	13 (26%)	9 (24%)	22 (25%)
25–54 *n* (%)	20 (39%)	26 (68%)	46 (51%)
ESBL-PE carriers (%)	51%	38%	44.5%

### Bacterial identification and antimicrobial susceptibility testing

The identified ESBL-PE strains included *Escherichia coli* (*n* = 64; *n* = 35 isolates from hospitalized patients and *n* = 29 from healthy volunteers), *Klebsiella pneumoniae* (*n* = 16; *n* = 11 isolates from hospitalized patients and *n* = 5 from healthy volunteers), *Enterobacter cloacae* (*n* = 6), *Citrobacter freundii* (*n* = 2), and *Morganella morganii* (*n* = 1) (Table 2).

**Table 2:** Fecal carriage rate of ESBL-PE among hospitalized patients and healthy volunteers

Strain	Hospitalized patients (*n* = 51)	Healthy volunteers (*n* = 38)	Total
*Escherichia coli n* (%)	35 (69%)	29 (76%)	64 (72%)
*Klebsiella pneumoniae n* (%)	11 (21%)	5 (13%)	16 (18%)
*Enterobacter cloacae n* (%)	5 (10%)	1 (3%)	6 (7%)
*Citrobacter freundii n* (%)	0	2 (5%)	2 (2%)
*Morganella morgani n* (%)	0	1 (3%)	1 (1%)

**Fig. 1:**
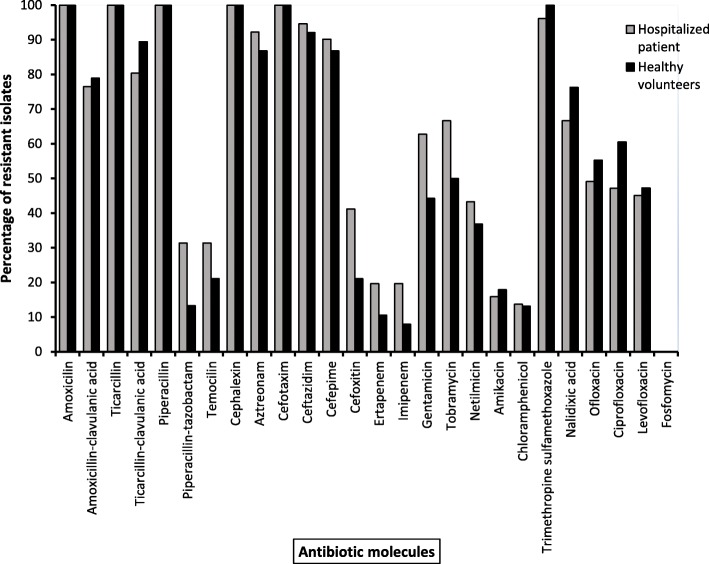
Percentage of antimicrobial resistance of ESBL-PE from healthy volunteers (*n* = 38) and hospitalized patients (*n* = 51) (X: antibiotic molecules, Y: percentage of resistant isolates). ESBL-PE, extended-spectrum ß-lactamase-producing *Enterobacteriaceae*

Antimicrobial susceptibility testing showed that few isolates were resistant to carbapenems (ertapenem and imipenem), particularly among hospitalized patients (Fig. 1). Resistance to aminoglycosides (gentamicin, tobramycin and netilmicin) was relatively high among hospitalized patients. Conversely, resistance to quinolones (nalidixic acid, ofloxacin, ciprofloxacin and levofloxacin) was higher among healthy volunteers. Most isolates were also resistant to sulfamethoxazole–trimethoprim and all isolates were susceptible to fosfomycin.

### Characterization of β-lactamase-encoding genes

Molecular characterization of the ESBL-PE isolates revealed that they harbored different ß-lactamase-encoding genes (*bla*_CTX-M_, *bla*_TEM_ and *bla*_OXA_). *bla*_CTX-M_ genes were detected in 97.8% (87/89) of ESBL-PE isolates. The distribution of the different ß-lactamase-encoding genes in the different *Enterobacteriaceae* species from hospitalized patients and healthy volunteers is shown in Table 3.

**Table 3:** ß-lactamase-encoding genes carried by the 89 ESBL-PE isolates from hospitalized patients **(***n* = 51) and healthy volunteers (*n* = 38)

Species	CTX-M-15	CTX-M-14	TEM	CTX-M-15/TEM	CTX-M-15/OXA-1	CTX-M-14/OXA-1	CTX-M-15/ TEM/OXA-1
Hospitalized patients							
*Escherichia coli n* = 35	14 (40%)	0	0	1 (3%)	10 (28%)	1 (3%)	9 (25%)
*Klebsiella pneumoniae n* = 11	3 (27%)	0	0	0	8 (73%)	0	0
*Enterobacter cloacae n* = 5	1 (20%)	0	1 (20%)	0	2 (40%)	1 (20%)	0
Healthy volunteers							
*Escherichia coli n* = 29	13 (49%)	2 (7%)	0	1 (3%)	13 (49%)	0	0
*Klebsiella pneumoniae n* = 5	3 (60%)	1 (20%)	0	0	1 (20%)	0	0
*Enterobacter cloacae n* = 1	1 (100%)	0	0	0	0	0	0
*Citrobacter freundii n* = 2	1 (50%)	0	0	0	1 (50%)	0	0
*Morganella morgana n* = 1	0	0	1 (100%)	0	0	0	0
Total *n* = 89	36 (40%)	3 (3%)	2 (2%)	2 (2%)	35 (39%)	2 (2%)	9 (10%)

Sequence analysis showed that among the 87 *bla*_CTX-M_-positive isolates, 82 contained *bla*_CTX-M-15_ and 5 carried *bla*_CTX-M-14_ alone or in combination with *bla*_TEM_ and *bla*_OXA_ (Table 3). The *bla*_OXA-1_ and bla_TEM_ genes were detected in 51.7% (46/89) and 14.6% (13/89) of isolates, respectively. The *bla*_CTX-M-15,_ *bla*_CTX-M-14_ and *bla*_TEM_ genes were detected alone in 36, 3 and 2 isolates respectively, whereas *bla*_CTX-M-15_, *bla*_OXA-1_ and *bla*_TEM_ were associated in 9 isolates. Moreover, 35 isolates coproduced *bla*_CTX-M-15_ and *bla*_OXA-1_, 2 harbored *bla*_CTX-M-15_ and *bla*_TEM_, and 2 expressed *bla*_CTX-M-15_ and *bla*_OXA-1_.

### Phylogenetic grouping

The majority of the 64 ESBL-producing *E. coli* isolates belonged to the phylogenetic groups A 17/64 (27%) and C 17/64 (27%), followed by groups B2 9/64 (14%), B1 8/64 (13%), D 8/64 (13%), and groups E and F (one isolate/each), The phylogenetic group of three isolates could not be classified according to Clermont *and al.* method [22] Table 4. The pandemic *E. coli* ST131 clone was identified in 100% (9/9) of the B2 group isolates. The carriage rate of B2-ST131 *E. coli* isolates among hospitalized patients and healthy volunteers was 11.42% (4/35) and 17.24% (5/29), respectively. Isolates classified in group C and D were more frequent among hospitalized patients than heathy volunteers (12% vs 5, and 6% vs 2%, respectively).

**Table 4:** Phylogenetic groups of 64 ESBL-producing *E. coli* isolates in hospitalized patients and healthy volunteers

	Phylogenetic groups								
	A	B1	B2	C	D	E	F	ND	ST131
Hospitalized patients *n* = 35	8 (23%)	4 (11%)	4 (11%)	12 (34%)	6 (17%)	1 (3%)	0	0	4
Healthy volunteers *n* = 34	9 (26%)	4 (12%)	5 (15%)	5 (15%)	2 (6%)	0	1 (3%)	3 (9%)	5
Total *n* = 64	17 (27%)	8 (13%)	9 (14%)	17 (27%)	8 (13%)	1 (1%)	1	3 (4%)	9

## Discussion

Fecal carriage of ESBL-PE isolates is one of the main drivers for their dissemination in hospital and community settings worldwide, but has received little attention in Africa. Our study shows high rate (45%) of ESBL-PE fecal carriage in hospitalized patients and also healthy volunteers in Chad. Our results confirm the high intestinal carriage of ESBL-PE in Sub-Saharan Africa reported in Cameroon (54%) and Burkina Faso (32%) [24, 25]. Among ESBL-PE isolates, resistance was associated predominantly with CTX-M15 genes. ESBL-producing *E. coli* isolates belonged to different phylogroups (mainly to the commensal phylogenetic groups A and B1). Our findings confirm the widespread dissemination of ESBL-encoding genes among different *Enterobacteriaceae* species isolated in the community and also in hospital.

ESBL-PE carriage was significantly higher in hospitalized patients compared with healthy volunteers (51% vs. 38%, *p* < 0.05), as previously described in similar settings [25–28]. This result could be explained by higher antibiotic consumption that favors ARB selection and/or higher rate of in-hospital acquisition of ARB that then colonize the patient’s intestinal track [29–31]. Several factors can contribute to high ARB selection in low income countries, particularly poor drug quality or inadequate posology, long treatments, antibiotic misuse by health professionals, unskilled practitioners, auto-medication (antibiotics can be purchased without prescription), unhygienic conditions accounting for the spread of resistant bacteria, and inadequate surveillance programs [9, 32, 33]. Future studies should evaluate the specific risk factors contributing to the observed high ESBL-PE level in Chad.

In this study, most ESBL-PE isolates were multidrug-resistant (i.e., resistant to three or more classes of antimicrobials) [34]. Other antibiotics included mainly quinolones, aminoglycosides (except amikacin), and co-trimoxazole (trimethoprim/sulfamethoxazole), as previously reported in Burkina Faso and Gabon [25, 35]. This could be explained by the presence of multiple resistance genes on the same plasmid [28, 36]. For example, quinolones and co-trimoxazole are often used to treat some mild to moderate infections (e.g., urinary tract infections) caused by *Enterobacteriaceae* in various clinical and community settings without previous laboratory testing of the bacteria involved in the infection [37].

Our study shows that the *bla*_CTX-M_ genes were the most common ß-lactamase-producing genes (98.8%) in the identified ESBL-PE isolates, with the predominance of *bla*_CTX-M-15_, in agreement with the reported worldwide spread of CTX-M-15 in community and hospital settings [3, 38]. Our finding also confirms the frequent association between *bla*_CTX-M-15_ and *bla*_OXA-1_ and/or *bla*_TEM_ genes in ESBL-PE isolates, which reduces the therapeutic options for treatment with β-lactam antibiotics [25, 28]. Moreover, the presence of the *bla*_CTX-M-14_ gene, which is predominant in Asian countries [39] but very rare in Chad, could be due to migration from Chad to China for medical treatment and economic reasons.

Molecular analysis revealed that the ESBL-producing *E. coli* isolates belonged to several phylogenetic groups, mainly to the commensal phylogenetic groups A and B1, the virulent extra-intestinal phylogenetic groups B2 and D, and group C. This finding is consistent with previous studies that showed a high phylogenetic diversity among ESBL-producing *E. coli,* and supports the hypothesis of a frequent horizontal gene transfers between distant *E. coli* phylogroups [3]. Moreover, the human pandemic O25b-ST13 clone was identified in all ESBL-producing *E. coli* from phylogroup B2 (9/9). ST131 is the most prevalent clone among the group B2 *E. coli* and is known to colonize the human digestive tract of healthy subjects [40].

Overall, the high carriage frequency of different ESBL-producing *Enterobacteriaceae* and *E. coli* strains suggest that ESBL diffusion is not caused by the epidemic spread of a single resistant clone, as previously found for extra-intestinal *E. coli* belonging to group B2 [41, 42].

## Conclusions

To our knowledge, this is the first study on the intestinal carriage of ESBL-PE in hospitalized patients and healthy community volunteers in Chad, and shows high carriage rate associated with the CTX-M-15 enzyme. The intestinal carriage of ESBL-PE is a significant challenge for public health, and highlights the urgent necessity to improve sanitation and implement antibiotic stewardship in African countries. Future studies should explore mechanisms involved in plasmid transfer and the determinants of the observed intestinal carriage.

## Data Availability

All data generated or analyzed during this study are included in this published article.
